# The measurements to reduce the rate of surgical site infection in a tertiary teaching hospital

**DOI:** 10.1186/2047-2994-4-S1-P84

**Published:** 2015-06-16

**Authors:** PW Yang, MH Hsieh, MC Chen, FY Tasia, JW Huang, CT Hung, PS Shie, CY Lin, YH Chen

**Affiliations:** 1 Department of Infection Control, Kaohsiung Medical University Hospital, Kaohsiung Medical University, Kaohsiung, Taiwan, Province of China; 2 Division of Infectious Diseases, Department of Internal Medicine, Kaohsiung Medical University Hospital, Kaohsiung Medical University, Kaohsiung, Taiwan, Province of China; 3 Department of Nursing, Kaohsiung Medical University Hospital, Kaohsiung Medical University, Kaohsiung, Taiwan, Province of China; 4 Division of Hepato-biliary-Pancreatic Surgery, Kaohsiung Medical University Hospital, Kaohsiung Medical University, Kaohsiung, Taiwan, Province of China; 5 Department of Medical Quality Control, Kaohsiung Medical University Hospital, Kaohsiung Medical University, Kaohsiung, Taiwan, Province of China

## Introduction

The surgical technique and equipment are much improved. However, surgical site infection(SSI) is still an important issue in medical care. SSI will increase length of stay, healthcare expense and workload of healthcare workers (HCWs).

## Objectives

In the Department of Hepatobiliary Surgery in a tertiary teaching hospital, the rate of SSI was 0.94% (January 2014 to April 2104), and increased to 4.46% (May 2014 to June 2014). In order to decrease the rate of SSIs, some measurements were applied.

## Methods

The factors may contribute the increased rate of SSI, which including no consensus in use of antimicrobial prophylaxis for surgery, no identical method of wound care, and the insufficient implementation of hand hygiene. After discussion some measurements were applied. First, the chief resident prescribed the antibiotic before surgery based on the guideline. Second, we revised the standard procedure of wound care. The procedure was announced to the HCWs and supervised by nurse practitioners and infection control nurses.

## Results

After introducing these measurements, the rate of surgical site infection decreased to 0.93% at the period of August 2014 to January 2015, compared to 4.46% at the period of May 2014 to June 2014. Our results show that implementation can reduce surgical site infection incidence.

## Conclusion

Surgical site infection is a common complication of surgery, which may reduce the quality of life, even leading to morbidity or mortality. Healthcare workers should had consensus in care of surgical wound and execute it carefully, in order to prevent further healthcare associated infection. After the teamwork with healthcare workers, the rate of surgical site infection reduced. The quality of care in surgical wound improved in the Department of Hepatobiliary Surgery.

## Disclosure of interest

None declared.

